# Post-translational modifications of PLK1: a dynamic regulatory network in cell cycle regulation and tumor progression

**DOI:** 10.3389/fcell.2026.1987587

**Published:** 2026-10-23

**Authors:** Wenjing Li, Huiping Lin, Donghua Wen

**Affiliations:** 1 Clinical Laboratory, Children’s Hospital Affiliated to Shandong University, Jinan, China; 2 Clinical Laboratory, Jinan Children’s Hospital, Jinan, China; 3 Department of Laboratory Medicine, Shanghai East Hospital, Tongji University School of Medicine, Shanghai, China

**Keywords:** phosphorylation, polo-like kinase 1, post-translational modifications, SUMOylation, tumor progression, ubiquitination

## Abstract

PLK1 is a highly conserved serine/threonine kinase that plays a central role in mitosis and cell-cycle progression. PLK1 is dynamically regulated by multiple post-translational modifications (PTMs), which coordinate its kinase activity, protein stability, subcellular localization, and protein–protein interactions, thereby ensuring its precise functions during cell-cycle progression and tumor progression. Despite substantial advances in understanding the post-translational regulation of PLK1, how different PTMs interact and coordinately regulate PLK1 remains incompletely understood. In this review, we systematically summarize the major PTMs of PLK1 and the molecular mechanisms through which they regulate PLK1 function, with particular emphasis on their roles in cell-cycle regulation and tumor progression. Beyond discussing individual PTMs, we integrate emerging evidence for crosstalk among different modifications, highlighting their cooperative, antagonistic, and sequential regulatory relationships and providing an integrated perspective on the dynamic post-translational regulation of PLK1. Finally, we discuss the biological significance of PLK1 PTM crosstalk, key unresolved questions in the field, and its potential implications for future cancer research and PLK1-targeted strategies.

## 1 Introduction

Ordered progression of the cell cycle is essential for maintaining genomic stability and ensuring faithful cell division (Matthews et al., 2022). Polo-like kinase 1 (PLK1), a highly conserved serine/threonine kinase, is a master regulator of mitosis and reaches peak expression during the G2/M phase. Through phosphorylation of numerous substrates, PLK1 orchestrates multiple mitotic events, including mitotic entry, centrosome maturation, spindle assembly, chromosome segregation, and cytokinesis (Joukov and De Nicolo, 2018; Kettenbach et al., 2011; Fu and Wen, 2017). Consequently, precise spatiotemporal regulation of PLK1 is indispensable for faithful cell-cycle progression and genome integrity.

Aberrant PLK1 expression has been reported in a wide range of human malignancies, including prostate, ovarian, small-cell lung, and pancreatic cancers, where it is frequently associated with aggressive clinicopathological features and poor patient outcomes (Fu and Wen, 2017; Iliaki et al., 2021). As a central regulator of mitosis, dysregulated PLK1 disrupts chromosome segregation and mitotic fidelity, thereby promoting chromosomal instability (CIN), aneuploidy, and tumor progression (Gheghiani et al., 2021). These findings establish PLK1 as not only a key regulator of cell-cycle progression but also a critical driver of tumorigenesis and cancer progression.

Post-translational modifications (PTMs) represent versatile regulatory mechanisms that dynamically modulate protein activity, stability, subcellular localization, and protein–protein interactions, thereby enabling rapid and reversible cellular responses to diverse physiological cues (Kumar et al., 2020). Recent reviews have further highlighted the critical roles of dysregulated PTM networks in cancer progression and their potential relevance as biomarkers and therapeutic targets (Gao et al., 2026; Bruno et al., 2025). PLK1 undergoes multiple PTMs, including phosphorylation, ubiquitination, small ubiquitin-like modifier (SUMO) conjugation (SUMOylation), methylation, and O-GlcNAcylation (Joukov and De Nicolo, 2018; Feldman et al., 2019; Yan et al., 2023; Liu and Zhang, 2017; Feitosa et al., 2018). Collectively, these PTMs coordinate PLK1 activation, kinase activity, protein stability and turnover, and cell-cycle progression, thereby ensuring the precise spatiotemporal regulation of PLK1 throughout the cell cycle.

Although considerable progress has been made in characterizing individual PTMs of PLK1, current knowledge remains largely fragmented, and how these modifications collectively coordinate PLK1 regulation is only beginning to emerge. Rather than acting as isolated regulatory events, distinct PTMs increasingly appear to cooperate, counteract one another, or occur in a defined sequence, collectively fine-tuning PLK1 activity in different cellular contexts. Integrating these findings is therefore essential for a comprehensive understanding of PLK1 regulation.

In this review, we systematically summarize the currently identified PTMs of PLK1 and their underlying molecular mechanisms, with particular attention to emerging evidence for PTM crosstalk and its roles in cell-cycle progression and tumor progression. We further discuss how an integrated understanding of PLK1 post-translational regulation may provide new perspectives for future studies of PLK1 biology and PTM-based therapeutic strategies in cancer.

## 2 Overview

### 2.1 Discovery and structural features of PLK1

The *Polo* gene was first identified in *Drosophila melanogaster*, and homologous proteins were subsequently discovered in *Saccharomyces cerevisiae* and *Xenopus laevis*, highlighting the evolutionary conservation of the Polo kinase family across eukaryotes (Lane and Nigg, 1997). In humans, the Polo-like kinase family comprises five members (PLK1–PLK5), among which PLK1 is the best-characterized and serves as a central regulator of cell-cycle progression (de Cárcer et al., 2011; Winkles and Alberts, 2005).

PLK1 consists of an N-terminal serine/threonine kinase domain (KD) and a C-terminal Polo-box domain (PBD) (Elia et al., 2003a). The PBD is composed of two conserved Polo-box motifs (PB1 and PB2) that fold into a functional phosphopeptide-binding module responsible for substrate recognition and subcellular targeting. It specifically recognizes phosphorylated serine/threonine-containing motifs with the consensus binding sequence Ser-[pSer/pThr]-[Pro/X], where X represents any amino acid (Elia et al., 2003a; Elia et al., 2003b). Through PBD-mediated substrate docking, PLK1 is recruited to distinct subcellular structures, thereby facilitating substrate phosphorylation by the kinase domain and ensuring precise spatiotemporal regulation of diverse mitotic events (Lowery et al., 2005; Kachaner et al., 2017).

### 2.2 Biological functions of PLK1

Orderly cell-cycle progression relies on the coordinated action of multiple cell-cycle regulators, among which PLK1 serves as a central kinase governing the G2/M transition and mitotic progression. PLK1 reaches peak expression during the G2/M phase and coordinates mitosis through the phosphorylation of a broad spectrum of substrates, thereby regulating virtually every stage of mitotic progression (Archambault and Glover, 2009). PLK1-dependent phosphorylation is required for mitotic entry, centrosome maturation, spindle assembly, chromosome segregation, and cytokinesis (Li et al., 2019; Bruinsma et al., 2012; Wang et al., 2008; Petronczki et al., 2008). PLK1 orchestrates distinct mitotic events through stage-specific substrate phosphorylation. During mitotic entry, PLK1 promotes cyclin dependent kinase 1 (CDK1) activation by phosphorylating cyclin B and cell division cycle 25C (CDC25C). It subsequently phosphorylates microtubule-associated proteins, including ninein-like protein (NLP), translationally controlled tumor protein (TCTP), and oncoprotein 18 (Op18), to facilitate centrosome maturation and spindle microtubule assembly (Budde et al., 2001; Casenghi et al., 2003; Yarm, 2002). During metaphase and anaphase, PLK1 targets centromere- and kinetochore-associated proteins, such as budding uninhibited by benzimidazole-related 1 (BUBR1), polo-box-interacting protein 1 (PBIP1), and polo-like kinase 1-interacting checkpoint helicase (PICH), to ensure bipolar spindle formation, accurate chromosome alignment, and faithful sister chromatid segregation (Kang et al., 2006; Baumann et al., 2007; Elowe et al., 2007). Through the spatiotemporally controlled phosphorylation of these substrates, PLK1 coordinates orderly mitotic progression while preserving chromosome segregation fidelity and genomic stability.

### 2.3 Post-translational modifications of PLK1

PTMs are reversible or irreversible covalent modifications that occur after protein synthesis. By adding or removing phosphate groups, ubiquitin, SUMO protein, methyl groups, and glycans, PTMs dynamically regulate protein activity, stability, subcellular localization, and protein–protein interactions, thereby governing diverse biological processes, including cell proliferation, differentiation, metabolism, and stress responses (Kumar et al., 2020; Walsh et al., 2005).

To date, PLK1 has been reported to undergo multiple PTMs, including phosphorylation, ubiquitination, SUMOylation, methylation, and O-GlcNAcylation (Joukov and De Nicolo, 2018; Feldman et al., 2019; Yan et al., 2023; Liu and Zhang, 2017; Feitosa et al., 2018). Collectively, these PTMs regulate PLK1 activity, stability, subcellular localization, substrate recognition, and protein turnover, thereby coordinating its diverse functions during cell-cycle progression and tumor progression (Joukov and De Nicolo, 2018; Feldman et al., 2019; Yan et al., 2023; Liu and Zhang, 2017; Feitosa et al., 2018).

Rather than acting independently, these PTMs increasingly appear to cooperate, antagonize one another, or occur in a sequential manner, collectively forming a dynamic regulatory network that fine-tunes PLK1 function during cell-cycle progression and tumor progression.

## 3 Phosphorylation and dephosphorylation of PLK1

Phosphorylation is the best-characterized post-translational modification regulating PLK1 function. As a reversible regulatory mechanism mediated by protein kinases and phosphatases, phosphorylation dynamically controls protein activity, conformation, stability, and protein–protein interactions, thereby playing fundamental roles in cellular signaling and cell-cycle regulation (Humphrey et al., 2015; Castelo-Soccio et al., 2023; Shi, 2009).

In addition to phosphorylating numerous downstream substrates during mitosis, PLK1 itself is subject to dynamic phosphorylation and dephosphorylation at multiple regulatory sites. These site-specific modifications collectively determine PLK1 kinase activity, subcellular localization, and substrate recognition, thereby regulating mitotic progression, DNA damage responses, and tumor progression.

### 3.1 Phosphorylation-dependent activation of PLK1

#### 3.1.1 Early studies on phosphorylation-dependent PLK1 activation

Early insights into the phosphorylation-dependent regulation of PLK1 were primarily derived from studies of its *X. laevis* homolog, Plx1. *Xenopus* Polo-like kinase kinase-1 (xPlkk1) was shown to directly activate Plx1 both *in vitro* and *in vivo*, thereby promoting the G2/M transition in oocytes and providing the first evidence that phosphorylation is essential for Polo-like kinase activation (Qian et al., 1998). Subsequent studies identified several phosphorylation sites in Plx1, including T201, S340, S326, S128, and S25/26 (Kelm et al., 2002). Among these, phosphorylation of T201 was established as the critical event required for Plx1 activation, whereas phosphorylation of S340 was associated with the characteristic mitotic electrophoretic mobility shift (Kelm et al., 2002).

Phosphomimetic analyses further demonstrated that the S128D/T201D double mutant markedly enhanced Plx1 kinase activity and promoted the activation of CDC25C and the Cyclin B–CDK1 complex (Qian et al., 1999; Ciardo et al., 2020). Notably, S326 in Plx1 corresponds to S335 in mammalian PLK1, indicating that phosphorylation-dependent regulation is highly conserved throughout the evolution of the Polo-like kinase family (Wind et al., 2002). In addition, protein kinase A (PKA) has been reported to phosphorylate PLK1 both *in vitro* and in HeLa cells and may contribute to meiotic progression in mouse oocytes (Shen et al., 2010). Collectively, these pioneering studies established site-specific phosphorylation as a fundamental mechanism underlying Polo-like kinase activation and laid the groundwork for subsequent characterization of the regulatory phosphorylation sites and activation mechanisms of mammalian PLK1.

#### 3.1.2 Mechanisms regulating PLK1 phosphorylation

T210 and S137 are the two major regulatory phosphorylation sites of PLK1, with phosphorylation of T210 being widely recognized as the pivotal molecular event required for PLK1 activation. The Aurora A–Bora complex constitutes the core regulatory module governing this activation process. During the G2 phase, CDK1 enhances Aurora A kinase activity by promoting its association with Bora, enabling Aurora A to phosphorylate PLK1 at T210 and thereby trigger PLK1 activation (Joukov and De Nicolo, 2018; Macůrek et al., 2008). In late G2, T210 phosphorylation further suppresses Bora-induced PLK1 dimerization, facilitating dimer dissociation into the catalytically active monomeric form and licensing mitotic entry (Raab et al., 2022).

Beyond the canonical Aurora A–Bora axis, multiple kinases and regulatory factors further promote PLK1 activation by enhancing or maintaining T210 phosphorylation. Vaccinia-related kinase 2 (VRK2) directly phosphorylates T210, thereby disrupting the interaction between PLK1 and checkpoint with forkhead and ring finger domains (CHFR) and antagonizing CHFR-mediated K209 ubiquitination and subsequent protein degradation, resulting in increased PLK1 stability (Zhu et al., 2021). In addition, SHC protein SH2 structural domain binding protein 1 (SHCBP1) enhances Aurora A-dependent T210 phosphorylation by binding to the PBD of PLK1, thereby promoting PLK1 activation (Tang et al., 2025). Receptor for activated C kinase 1 (RACK1) facilitates the recruitment of Aurora A to centrosomes, increasing centrosomal T210 phosphorylation of PLK1 and contributing to centrosome duplication during S phase (Yoshino et al., 2020).

As cells progress into mitosis, Bora is progressively degraded, rendering the Aurora A–Bora complex insufficient to sustain maximal PLK1 activity. At this stage, checkpoint kinase 2 (CHK2) functions as a key kinase that directly phosphorylates T210 to maintain PLK1 activation (Black et al., 2024). Collectively, these findings indicate that PLK1 activation is not governed by a single upstream kinase but is orchestrated by multiple kinases and regulatory proteins that coordinate T210 phosphorylation in a cell cycle-dependent manner, thereby ensuring precise temporal control of PLK1 activity during mitosis.

Beyond the canonical Aurora A–Bora axis, multiple signaling pathways and regulatory factors contribute to PLK1 activation. The tumor suppressor disabled homolog 2-interacting protein (DAB2IP) promotes PLK1 activation by interacting with PLK1 through its CPR domain and facilitating T210 phosphorylation (Yu et al., 2016). In addition, the PIM2–CHK1–PLK1 signaling cascade represents an alternative activation pathway independent of Aurora A–Bora. In this pathway, pim-2 proto-oncogene, serine/threonine kinase (PIM2) phosphorylates CHK1 at S280, enabling CHK1 to phosphorylate PLK1 at T210 and thereby promote PLK1 activation (Adam et al., 2018).

In addition to T210, phosphorylation at S137 also contributes to the fine regulation of PLK1 activity. During the DNA damage response, CHK1 phosphorylates PLK1 at S137, thereby reducing its interaction with poly (ADP-ribose) (PAR) and preventing its recruitment to sites of DNA damage while enhancing its kinase activity toward RAD51 recombinase (RAD51), highlighting a regulatory role for S137 phosphorylation in PLK1-mediated DNA damage signaling (Peng et al., 2021). Furthermore, following CDK1-dependent phosphorylation, the adaptor protein WW domain-containing adaptor with coiled-coil (WAC) binds to the polo-box domain (PBD) of PLK1, thereby promoting PLK1 activation and enhancing downstream signaling (Qi et al., 2018).

Collectively, these studies demonstrate that PLK1 phosphorylation is coordinated by the Aurora A–Bora axis together with multiple upstream kinases and regulatory factors. Dynamic phosphorylation of key regulatory residues, including T210 and S137, collectively fine-tunes the spatiotemporal regulation of PLK1 activity.

### 3.2 T210- and S137-mediated regulation of the cell cycle

Phosphorylation of T210 and S137 represents the two most critical regulatory events controlling PLK1 function during mitosis. Among these, phosphorylation at T210 is widely recognized as the pivotal event required for PLK1 activation and precedes phosphorylation at S137. S137 phosphorylation has been detected during late mitosis, although whether it occurs specifically during anaphase or cytokinesis remains unresolved, and contributes to the subsequent fine-tuning of PLK1 activity (Tsvetkov and Stern, 2005; van de Weerdt et al., 2005). Although phosphorylation at both sites facilitates mitotic entry, they exert distinct regulatory functions during subsequent stages of mitotic progression.

Genetic studies have further delineated the functional specialization of T210 and S137 phosphorylation at different stages of mitosis. Phosphorylation of T210 is primarily involved in G2/M transition, centrosome maturation, spindle assembly, and spindle assembly checkpoint (SAC) regulation. The phospho-mimetic mutant T210D promotes mitotic entry and partially rescues the G2 arrest caused by Cyclin A2 depletion; however, it also induces SAC-dependent mitotic delay and prolonged M-phase progression. In contrast, the phosphorylation-deficient mutant T210A results in monopolar spindle formation and mitotic arrest (Tsvetkov and Stern, 2005; van de Weerdt et al., 2005; Jang et al., 2002; Silva Cascales et al., 2021; Bruinsma et al., 2014). Consistent with this role, RACK1-dependent T210 phosphorylation contributes to centrosome maturation and duplication during S phase, further highlighting the importance of T210 phosphorylation in centrosome function (Yoshino et al., 2020). Moreover, sustained T210-dependent PLK1 activity is required for accurate chromosome alignment, faithful chromosome segregation, and successful cytokinesis, thereby preserving genome stability (Black et al., 2024). In addition, T210-dependent PLK1 signaling maintains spindle assembly checkpoint integrity, chromosome stability, and DNA replication initiation through the PLK1–MPS1 and PLK1–HBO1 pathways (Shang et al., 2025; Yu et al., 2022). Collectively, these findings demonstrate that T210 phosphorylation is not only indispensable for PLK1 activation but also serves as a central regulatory event coordinating cell-cycle progression, spindle dynamics, and genome stability.

In contrast, phosphorylation at S137 is primarily associated with centrosome function and the regulation of mitotic progression. Expression of the phospho-mimetic S137D mutant or the S137D/T210D double mutant results in untimely APC/C activation and frequent mitotic catastrophe (van de Weerdt et al., 2005). In a separate study, expression of S137D arrested HeLa cells in early S phase with slightly separated centrosomes (Jang et al., 2002). In the DNA damage response, CHK1-mediated phosphorylation of PLK1 at S137 not only suppresses its binding to PAR and prevents its recruitment to DNA double-strand breaks (DSBs), but also maintains T210 phosphorylation and PLK1 kinase activity toward RAD51 at S14, thereby facilitating homologous recombination-mediated DNA repair (Peng et al., 2021). Consistent with these findings, disruption of S137-dependent PLK1 signaling impairs G2/M progression and chromosome alignment, further supporting its role in maintaining mitotic fidelity and genome stability (Qi et al., 2018). Collectively, these findings indicate that S137 phosphorylation not only regulates late mitotic events but also contributes to DNA damage signaling and chromosome stability.

Overall, phosphorylation at T210 and S137 regulates distinct stages of the cell cycle. Whereas T210 phosphorylation primarily governs PLK1 activation, mitotic entry, spindle assembly, and chromosome stability, S137 phosphorylation predominantly fine-tunes checkpoint signaling, mitotic progression, and the DNA damage response. Together, these complementary phosphorylation events ensure the spatiotemporal regulation of PLK1 activity required for faithful cell-cycle progression and maintenance of genome integrity.

### 3.3 Non-canonical phosphorylation-mediated regulation of PLK1 activity and function

#### 3.3.1 Tyrosine phosphorylation-mediated regulation of PLK1

In addition to the well-characterized serine/threonine phosphorylation events, PLK1 also undergoes tyrosine phosphorylation. To date, four tyrosine phosphorylation sites have been identified, including Y166, Y217, Y425, and Y445 (Wang et al., 2024; Caron et al., 2016; Yang et al., 2017; Liu et al., 2016). Unlike T210 and S137 phosphorylation, which directly regulate PLK1 activation, tyrosine phosphorylation primarily serves as a fine-tuning mechanism by modulating PLK1 conformation, kinase activity, and protein stability.

Protein kinase, membrane associated tyrosine/threonine 1 (PKMYT1) phosphorylates PLK1 at Y217, thereby suppressing phosphorylation of the activation-loop residue T210 and reducing PLK1 kinase activity (Caron et al., 2016). Although Y217 phosphorylation negatively regulates PLK1 activation, PKMYT1-dependent regulation of PLK1 expression and phosphorylation has also been implicated in the progression of pancreatic ductal adenocarcinoma (Wang et al., 2024). In addition, ABL proto-oncogene 1, non-receptor tyrosine kinase (c-Abl) phosphorylates PLK1 at Y425, Y217, and Y445, with Y425 representing the predominant phosphorylation site. Phosphorylation at Y425 induces a conformational change in PLK1 that enhances the Aurora A–PLK1 interaction, thereby promoting Aurora A-mediated phosphorylation of T210 while suppressing PLK1 ubiquitination and proteasomal degradation. These coordinated effects enhance PLK1 activity and facilitate timely mitotic entry (Yang et al., 2017). Consistently, reduced Y425 phosphorylation delays cell-cycle progression, whereas aberrant c-Abl-mediated tyrosine phosphorylation of PLK1 is associated with cervical cancer progression and poor patient prognosis (Yang et al., 2017). By contrast, Src proto-oncogene, non-receptor tyrosine kinase (c-Src) mediated phosphorylation of Y166 and Y445 suppresses PLK1 activity and is associated with impaired SAC function, increased chromosome misalignment, and mitotic abnormalities (Liu et al., 2016). Collectively, these findings indicate that tyrosine phosphorylation acts as an additional regulatory layer that fine-tunes PLK1 activity by coordinating kinase activation, protein stability, and mitotic fidelity.

#### 3.3.2 Additional regulatory phosphorylation sites

Beyond T210, S137, and the identified tyrosine phosphorylation sites, PLK1 contains several additional phosphorylation sites with distinct regulatory functions. PLK1 S99 phosphorylation occurs independently of T210 phosphorylation and largely depends on PI3K-AKT signaling. It promotes the recruitment of 14-3-3γ to PLK1, thereby enhancing PLK1 catalytic activity and facilitating proper metaphase–anaphase progression (Kasahara et al., 2013). In addition, phosphorylated p38 and MAP kinase-activated protein kinase 2 (MK2) colocalize with PLK1 at spindle poles during prophase and metaphase. MK2 directly phosphorylates PLK1 at S326, and disruption of S326 phosphorylation causes mitotic arrest, likely due to defective bipolar spindle formation and subsequent spindle checkpoint activation (Tang et al., 2008). Extracellular signal-regulated kinase (ERK) has been shown to phosphorylate PLK1 at T198, S404, S341, and S638, of which T198 and S404 represent canonical ERK phosphorylation sites, whereas S341 and S638 are classified as non-canonical sites (Baek et al., 2025). Functional studies have demonstrated that phosphorylation at S404 regulates nuclear dynamics during embryonic development by suppressing nuclear envelope breakdown and preserving nuclear integrity (Baek et al., 2025). Another functionally important phosphorylation site is T214. Although the upstream kinase responsible for T214 phosphorylation has not yet been identified, phosphorylation at this residue is required for the mitotic functions of PLK1. Accordingly, substitution of T214 with valine (T214 V) results in mitotic arrest and impaired cell proliferation, highlighting the essential role of this site in PLK1-mediated mitotic progression (Lasek et al., 2016).

Although the functions of these non-canonical phosphorylation sites have not been fully elucidated, current evidence suggests that PLK1 activity is coordinated by a multilayered phosphorylation network. Distinct phosphorylation events may confer context-dependent regulatory functions, thereby expanding the versatility of PLK1 in different physiological and cellular settings.

### 3.4 Functions of PLK1 phosphorylation beyond mitosis

Beyond mitosis, PLK1 phosphorylation also plays essential roles in other physiological processes. During meiosis, inhibition of PLK1 by BI2536 suppresses phosphorylation of S137 in PLK1 localized between microtubule-organizing centers (MTOCs) and chromosome arms, thereby impairing the recruitment of γ-tubulin and pericentriolar material, disrupting meiotic spindle assembly, delaying REC8 meiotic recombination protein (REC8) degradation, and ultimately causing chromosome misalignment during meiosis I in oocytes (Du et al., 2015). These findings demonstrate that S137 phosphorylation is essential for proper spindle assembly and faithful chromosome segregation during meiosis.

In addition to its role in cell-cycle regulation, T210 phosphorylation contributes to smooth muscle cell proliferation and contractile function by regulating cytoskeletal dynamics. The phosphorylation-deficient mutant T210A suppresses platelet-derived growth factor (PDGF) induced activation of the MEK1/2–ERK1/2 pathway, resulting in reduced smooth muscle cell proliferation (Jiang and Tang, 2015). Furthermore, depletion of nestin decreases T210 phosphorylation and disrupts cytoskeletal signaling (Wang et al., 2023). T210A also inhibits acetylcholine-induced phosphorylation of vimentin at S56, attenuates smooth muscle contraction, and impairs focal adhesion organization by weakening the interaction between vimentin filaments and paxillin (Wang et al., 2022; Li et al., 2016). These findings highlight a previously unrecognized role for T210 phosphorylation in coordinating cytoskeletal remodeling and smooth muscle function.

Aberrant PLK1 phosphorylation is also closely associated with tumor progression, with persistent phosphorylation at T210 representing a major oncogenic event. The phospho-mimetic mutant T210D induces epithelial–mesenchymal transition (EMT) and upregulates transforming growth factor beta (TGF-β) and tumor necrosis factor alpha-induced protein 6 (TSG6) expression, thereby promoting tumor cell migration, invasion, and tumorigenesis (Shin et al., 2020). In addition, several oncogenic regulators enhance T210 phosphorylation to further amplify PLK1 signaling. For example, the long non-coding RNA AFAP1-AS1 promotes T210 phosphorylation to drive mitotic progression in triple-negative breast cancer (TNBC) cells (Cen et al., 2023), whereas RGCC stabilizes the active conformation of PLK1 through simultaneous interactions with the KD and PBD, thereby enhancing T210 phosphorylation and facilitating lung metastasis of TNBC (Cheng et al., 2023). Collectively, these findings identify sustained T210 phosphorylation as a critical molecular event linking aberrant PLK1 activation to tumor progression and metastasis.

### 3.5 Mechanisms regulating PLK1 dephosphorylation

Counterbalancing phosphorylation-dependent PLK1 activation, multiple protein phosphatases negatively regulate PLK1 activity through site-specific dephosphorylation, thereby coordinating cell-cycle progression, DNA damage responses, and chromosome stability.

The MYPT1–PP1cβ complex represents one of the best-characterized mechanisms controlling PLK1 dephosphorylation. CDK1-dependent phosphorylation of myosin phosphatase target subunit 1 (MYPT1) promotes its interaction with the polo-box domain (PBD) of PLK1, thereby recruiting protein phosphatase 1 catalytic subunit beta (PP1cβ) to dephosphorylate T210 and suppress PLK1 kinase activity (Yamashiro et al., 2008; Archambault et al., 2008). Several upstream regulators, including CHK1, CHK2, LATS1, and NUAK1, modulate PLK1 activity by regulating MYPT1 phosphorylation (Nai et al., 2019; Meng et al., 2026; Chiyoda et al., 2012; Hu et al., 2018; Banerjee et al., 2014). In addition, O-GlcNAcylation of MYPT1 weakens its interaction with PLK1, leading to enhanced T210 phosphorylation and increased PLK1 kinase activity (Liu et al., 2020). Collectively, these findings identify MYPT1 as not only a key mediator of PLK1 dephosphorylation but also an important signaling hub integrating phosphorylation and O-GlcNAcylation to fine-tune PLK1 activity.

Another major phosphatase regulating PLK1 is protein phosphatase 2A (PP2A), together with its regulatory subunits B55α and B55δ (Wang et al., 2015; Jang et al., 2007). Following DNA damage, activation of the ATM/ATR–CHK signaling pathway promotes PP2A-dependent dephosphorylation and inactivation of PLK1, thereby preventing mitotic progression and chromosome segregation. Under certain conditions, PLK1 dephosphorylation can even drive cells from metaphase back to early mitosis or G2 phase (Wang et al., 2015; Jang et al., 2007; Rivera and Losada, 2006).

During recovery from severe DNA damage, PP2A–B55-mediated PLK1 dephosphorylation induces mitotic slippage, allowing damaged cells to exit mitosis without completing chromosome segregation or cytokinesis, thereby limiting the proliferation of genomically compromised cells (Kim et al., 2019). In addition, at kinetochores, PP1 and PP2A–B56 promote the removal of PLK1 from the BUB complex, terminating the MELT phosphorylation-dependent positive-feedback loop and facilitating SAC silencing (Cordeiro et al., 2020). Together, these findings establish PP2A-mediated dephosphorylation as a central mechanism coupling DNA damage checkpoints with mitotic exit and checkpoint silencing.

In addition to the canonical phosphatases described above, protein phosphatase 4 catalytic subunit (PPP4C), phosphatase and tensin homolog (PTEN), and EYA transcriptional coactivator (EYA) family also participate in the regulation of PLK1 dephosphorylation through distinct mechanisms. PPP4C suppresses PLK1 activity by dephosphorylating S137 and contributes to the repair of DNA double-strand breaks (Dong et al., 2022). PPP4C-mediated PLK1 inactivation is essential for RAD51-dependent homologous recombination, whereas sustained PLK1 activity disrupts the coordination between DNA damage checkpoint signaling and DNA repair (Dong et al., 2022).

Unlike PPP4C, PTEN indirectly restrains PLK1 activation by preventing excessive phosphorylation of both Aurora A and PLK1, thereby preserving chromosome stability and preventing chromosome mis-segregation during mitosis (Li W. et al., 2025; Ghosh et al., 2023; Zhang et al., 2016). Loss of PTEN confers resistance to spontaneous polyploidization and spindle disruption, further highlighting its role in maintaining mitotic fidelity (Li W. et al., 2025; Ghosh et al., 2023; Zhang et al., 2016).

In contrast, EYA1 and EYA4 directly dephosphorylate PLK1 at Y445, a modification that is critical for centrosome maturation, centrosomal localization, and PLK1 activation during G2 phase (Nelson et al., 2024). Phosphorylation of Y445 interferes with PBD-mediated substrate binding, whereas EYA-dependent dephosphorylation restores this interaction, thereby facilitating assembly of the PLK1 activation complex and promoting its biological functions (Nelson et al., 2024).

Collectively, these findings demonstrate that PLK1 dephosphorylation is not merely a mechanism for terminating kinase activity but also represents an essential regulatory process coordinating cell-cycle progression, DNA damage repair, chromosome segregation, and mitotic fidelity. Together with phosphorylation-dependent activation, reversible phosphorylation establishes a dynamic regulatory circuit that ensures the precise spatiotemporal control of PLK1 activity.

The major mechanisms by which phosphorylation regulates PLK1 activity and function in both mitotic and non-mitotic processes are summarized in Figure 1. A summary of the regulatory network of PLK1 phosphorylation is provided in Table 1.

**FIGURE 1:**
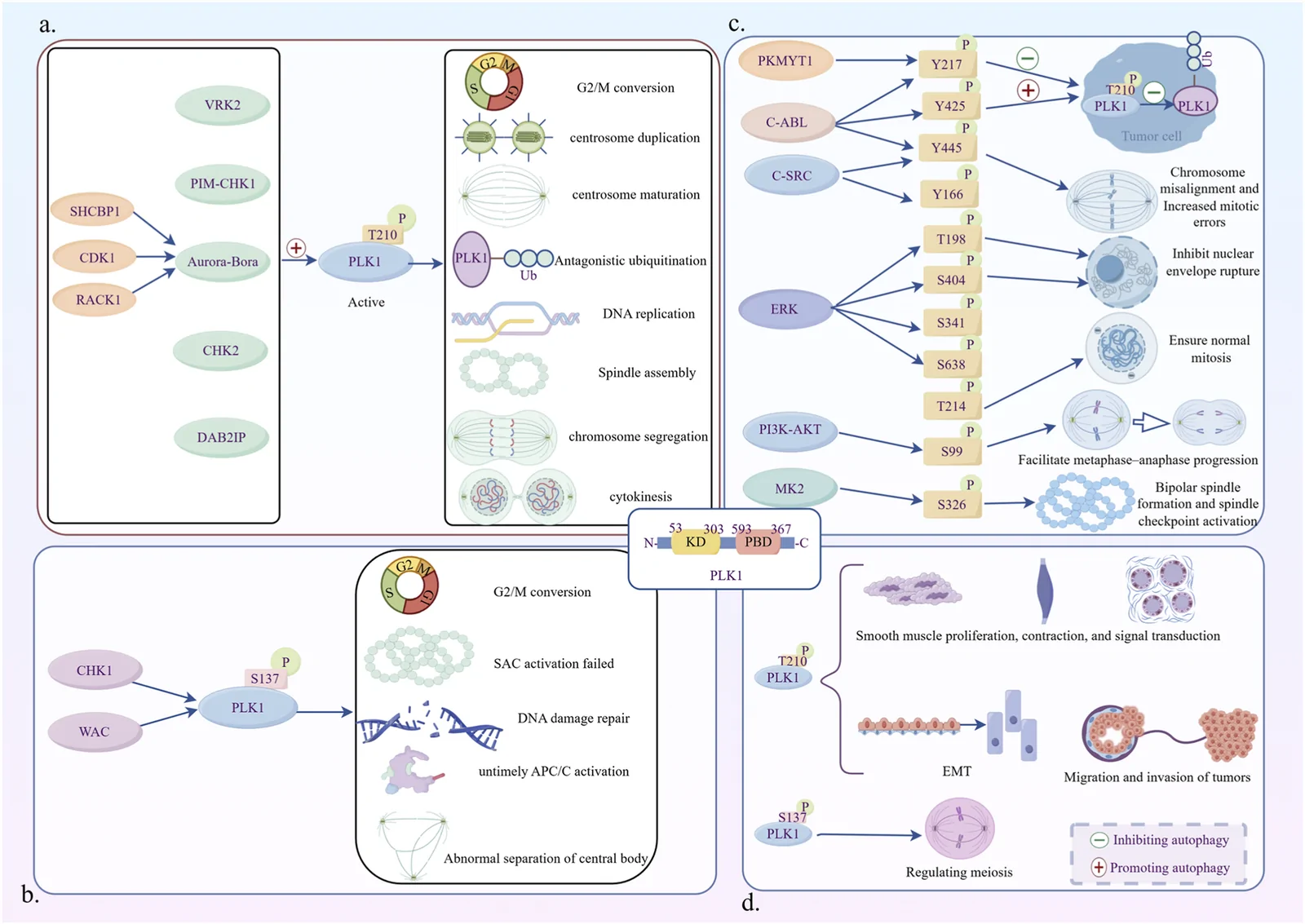
Phosphorylation-mediated regulation of PLK1 in mitotic and non-mitotic processes. Panels **(a**–**c)** summarize the effects of phosphorylation at different PLK1 residues on mitotic progression, whereas panel **(d)** illustrates the roles of PLK1 phosphorylation in non-mitotic biological processes. **(a)** Phosphorylation of Thr210 represents the major activation event of PLK1, enabling its kinase activity during mitosis. Activated PLK1 regulates G2/M transition, centrosome duplication, centrosome maturation, antagonistic ubiquitination, DNA replication, spindle assembly, chromosome segregation, and cytokinesis. Phosphorylation of Thr210 is directly mediated by VRK2, PIM–CHK1, Aurora A–Bora, CHK2, and DAB2IP, whereas SHCBP1, CDK1, and RACK1 indirectly promote Thr210 phosphorylation by facilitating the Aurora A–Bora signaling axis. **(b)** In addition to Thr210, phosphorylation of Ser137 is another critical regulatory event for PLK1 activation. CHK1 and WAC phosphorylate Ser137 to regulate G2/M transition, DNA damage repair, untimely APC/C activation, frequent mitotic catastrophe, and abnormal midbody separation during mitosis. **(c)** In addition to Thr210 and Ser137, PLK1 is phosphorylated at four tyrosine residues (Y166, Y217, Y425, and Y445) and five serine/threonine residues (T198, T241, S341, S404, and S638). PKMYT1-mediated phosphorylation of Y217 suppresses Thr210 phosphorylation, thereby weakening the antagonistic effect of Thr210 phosphorylation on PLK1 ubiquitination and promoting PLK1 ubiquitination in tumor cells. Conversely, phosphorylation of Y425 enhances Thr210 phosphorylation and reinforces its antagonistic effect on PLK1 ubiquitination, leading to PLK1 stabilization. c-Abl phosphorylates Y217, Y425, and Y445, whereas c-Src phosphorylates Y166 and Y445. Phosphorylation of Y445 induces chromosome misalignment and increases mitotic errors. ERK phosphorylates T198, S341, S404, and S638; phosphorylation of T198 and S404 inhibits nuclear envelope rupture, whereas phosphorylation of T241 is required for normal mitotic progression. S99 and S326 phosphorylation promote metaphase–anaphase progression and support bipolar spindle formation and mitotic progression, respectively. **(d)** Beyond mitosis, phosphorylation of Thr210 and Ser137 also regulates non-mitotic functions. Thr210 phosphorylation promotes smooth muscle proliferation, contraction and signal transduction, as well as epithelial–mesenchymal transition (EMT), tumor cell migration, and invasion. In contrast, Ser137 phosphorylation is involved in the regulation of meiosis (By Figdraw).

**TABLE 1:** Regulatory network of PLK1 phosphorylation and its biological significance.

Regulator	Target site	Regulatory role in PLK1 phosphorylation and biological significance
Phosphorylation regulators		
VRK2 (Zhu et al., 2021)	T210	Promotes T210 phosphorylation, antagonizes CHFR-mediated K209 ubiquitination, stabilizes PLK1, and facilitates G2/M progression
DAB2IP (Shang et al., 2025; Yu et al., 2022)	T210	Enhances T210 phosphorylation and activates PLK1, thereby maintaining spindle assembly checkpoint, chromosome stability, and DNA replication initiation
Aurora A–Bora (Joukov and De Nicolo, 2018; Macůrek et al., 2008; Raab et al., 2022)	T210	Induces T210 phosphorylation and activates PLK1
CHK2 (Black et al., 2024)	T210	Maintains T210 phosphorylation after Bora degradation to sustain PLK1 activity, ensuring proper chromosome alignment, chromosome segregation, and cytokinesis
PIM-CHK1 (Adam et al., 2018)	T210	Activates PLK1 through the PIM–CHK1–T210 signaling cascade, promoting mitotic entry and cell proliferation
CHK1 (Peng et al., 2021)	S137	Promotes S137 phosphorylation, prevents PAR binding and PLK1 recruitment to DSBs, maintains T210 phosphorylation, and preserves PLK1 kinase activity toward RAD51 Ser14 during DNA damage repair
WAC (Qi et al., 2018)	S137	Binds the PLK1 PBD following CDK1-dependent phosphorylation of WAC, promotes PLK1 activation, and facilitates G2/M transition and chromosome congression
PKMYT1 (Wang et al., 2024)	Y217	Phosphorylates Y217, inhibits T210 phosphorylation and PLK1 activation, and is associated with pancreatic ductal adenocarcinoma progression
c-Abl (Yang et al., 2017)	Y425, Y217, Y445	Phosphorylates Y425, Y217, and Y445, with Y425 as the predominant site; Y425 phosphorylation enhances Aurora A-mediated T210 phosphorylation, suppresses PLK1 ubiquitination and degradation, promotes PLK1 activation and mitotic entry, and is associated with cervical cancer progression and poor prognosis
c-Src (Liu et al., 2016)	Y166, Y445	Phosphorylates Y166 and Y445, suppresses PLK1 activity, and is associated with impaired mitotic checkpoint function, chromosome misalignment, and aberrant mitosis
PI3K-AKT (Kasahara et al., 2013)	S99	Enhances PLK1 activity via 14-3-3γ binding and promotes metaphase–anaphase transition
MK2 (Tang et al., 2008)	S326	Supports bipolar spindle formation and proper mitotic progression
ERK (Baek et al., 2025)	T198, S341, S404, S638	Phosphorylates multiple PLK1 residues, including canonical sites T198 and S404 and non-canonical sites S341 and S638; S404 phosphorylation regulates nuclear dynamics and maintains nuclear envelope integrity during embryonic development
Unknown kinase (Lasek et al., 2016)	T214	Phosphorylation at T214 is essential for PLK1 mitotic function, and loss of T214 phosphorylation causes mitotic arrest and proliferation defects
Dephosphorylation regulators		
PP1cβ (Yamashiro et al., 2008; Archambault et al., 2008)	T210	Dephosphorylates Thr210 and suppresses PLK1 activity
PP2A-B55 (Wang et al., 2015; Jang et al., 2007; Rivera and Losada, 2006; Kim et al., 2019)	\	Mediates PLK1 dephosphorylation after DNA damage, suppresses mitotic progression, and promotes mitotic slippage during recovery from severe DNA damage
PP2A-B56 (Cordeiro et al., 2020)	\	Promotes dephosphorylation and removal of BUB-associated PLK1 at kinetochores together with PP1, terminates the MELT phosphorylation positive-feedback loop, and facilitates spindle assembly checkpoint silencing
PPP4C (Dong et al., 2022)	S137	Dephosphorylates Ser137, suppresses PLK1 activity, and coordinates RAD51-dependent homologous recombination during DNA damage repair
PTEN (Li et al., 2025a; Ghosh et al., 2023; Zhang et al., 2016)	\	Restrains Aurora A- and PLK1 hyperphosphorylation, maintains chromosome stability, prevents chromosome missegregation, and protects against spontaneous polyploidization and spindle breakage
EYA (Nelson et al., 2024)	Y445	Dephosphorylates Y445, restores PBD-mediated substrate binding, promotes PLK1 activation complex assembly, and is required for centrosome maturation, centrosomal localization, and PLK1 activation during G2

This table summarizes the upstream kinases and phosphatases involved in regulating PLK1 phosphorylation and dephosphorylation, including their target modification sites, regulatory effects on PLK1 activity, and associated biological consequences during cell-cycle progression and tumor development.

## 4 PLK1 methylation

Protein methylation primarily occurs on lysine and arginine residues and plays essential roles in transcriptional regulation, DNA damage repair, and protein function (Waterborg, 1993; Park et al., 2022). Although methylation of the PLK1 promoter has been extensively investigated in cancer diagnosis and prognosis, studies on PLK1 protein methylation have emerged only recently (Deng et al., 2021; Zhang et al., 2019; Chen et al., 2025; Liu et al., 2025). To date, multiple lysine methylation sites have been identified in PLK1, including K191, K209, K284, K388, K413, K420, K474, K492, K540, and K574 (Feldman et al., 2019; Li et al., 2019; Yu et al., 2020). Current evidence indicates that PLK1 methylation primarily regulates kinase activity and cooperates with other post-translational modifications to fine-tune cell-cycle progression.

One of the major functions of PLK1 methylation is to restrain kinase activity. During mitosis, SET domain containing 6 (SETD6) methylates PLK1 at K209 and K413, thereby suppressing kinase activity and slowing mitotic progression. Conversely, preventing methylation at these sites enhances PLK1 catalytic activity, accelerates mitosis, and promotes cell proliferation (Feldman et al., 2019). Similarly, SET domain containing lysine methyltransferase 7 (SETD7, also known as SET7/9) catalyzes di-methylation of K191 during late G2 and mitosis, limiting ATP utilization and maintaining optimal PLK1 activity at kinetochores to facilitate correction of kinetochore–microtubule attachment errors (Yu et al., 2020). Together, these findings suggest that lysine methylation serves as an intrinsic mechanism preventing excessive PLK1 activation during mitosis.

In addition to cell-cycle regulation, PLK1 methylation also contributes to the DNA damage response. Euchromatic histone lysine methyltransferase 2 (EHMT2, commonly known as G9a) mediated mono-methylation of K209 peaks during S phase and is further enhanced following DNA damage (Li et al., 2019). Functionally, K209 methylation suppresses PLK1 activity to coordinate DNA replication and DNA repair, whereas the methylation-deficient mutant K209 A disrupts normal DNA replication (Li et al., 2019).

Notably, PLK1 methylation exhibits functional crosstalk with phosphorylation. G9a-mediated methylation of K209 and Aurora A-mediated phosphorylation of T210 are mutually antagonistic: methylation of K209 inhibits phosphorylation of T210 and suppresses PLK1 activation, whereas phosphorylation of T210 prevents methylation at K209 (Li et al., 2019). This methylation–phosphorylation switch provides an additional layer of regulation that dynamically fine-tunes PLK1 activity.

Collectively, PLK1 methylation functions as a negative regulatory mechanism that restrains kinase activity, coordinates DNA damage responses, and cooperates with phosphorylation to maintain cell-cycle progression and genome stability. Although multiple methylation sites have been identified, the biological functions of many of these modifications and their interplay with other post-translational modifications remain to be fully elucidated.

## 5 PLK1 O-GlcNAcylation

O-GlcNAcylation is a dynamic and reversible post-translational modification catalyzed by O-linked N-acetylglucosamine transferase (OGT) and has emerged as an important regulator of cell-cycle progression (Le et al., 2016; Wang et al., 2010). PLK1 has been identified as a substrate of OGT, with T291 serving as the primary O-GlcNAcylation site and T288 as a minor modification site (Yan et al., 2023; Wang et al., 2010; Li J. et al., 2025; Qin et al., 2017). Unlike the canonical role of O-GlcNAcylation in protecting proteins from ubiquitin-dependent degradation, O-GlcNAcylation of PLK1 promotes its ubiquitination, thereby facilitating PLK1 turnover and ensuring proper mitotic progression (Yan et al., 2023; Wang et al., 2010; Qin et al., 2017).

Functional studies further demonstrated that disruption of O-GlcNAcylation at T291 (T291A or T291N) results in mitotic delay and chromosome segregation defects, highlighting the essential role of this modification in maintaining chromosome stability and faithful cell division (Yan et al., 2023). Notably, both T291A and T291N mutants promote uterine tumor growth in mouse xenograft models (Yan et al., 2023), suggesting that dysregulated PLK1 O-GlcNAcylation contributes to tumorigenesis by impairing mitotic fidelity.

Collectively, these findings establish O-GlcNAcylation as an important regulator of PLK1 function that links protein turnover with chromosome stability. Its functional crosstalk with ubiquitination further expands the complexity of PLK1 post-translational regulation and highlights the importance of PTM coordination in controlling PLK1 activity.

## 6 Ubiquitination and deubiquitination of PLK1

### 6.1 PLK1 ubiquitination

Ubiquitination is a reversible post-translational modification in which ubiquitin is covalently conjugated to substrate proteins through the sequential actions of E1 activating enzymes, E2 conjugating enzymes, and E3 ligases. This modification regulates protein degradation, stability, localization, and signaling pathways (Squair and Virdee, 2022). The dynamic balance between ubiquitination and deubiquitination is essential for maintaining PLK1 function and plays critical roles in chromosome alignment, sister chromatid segregation, and cell-cycle progression (Liu and Zhang, 2017). To date, K492 and K209 have been identified as the major ubiquitination sites of PLK1, where distinct ubiquitin modifications regulate PLK1 subcellular localization, protein stability, spindle checkpoint signaling, and tumor progression.

One of the best-characterized functions of PLK1 ubiquitination is the regulation of kinetochore localization. To ensure faithful chromosome segregation, PLK1 is recruited to kinetochores during prometaphase and must be released before sister chromatid separation. Cullin 3 (CUL3)–kelch-like family member 22 (KLHL22)-mediated mono-ubiquitination of K492 disrupts the interaction between PLK1 and kinetochore phospho-receptors, thereby promoting PLK1 dissociation from kinetochores without affecting its overall cellular abundance (Beck et al., 2013; McGourty and Rape, 2013). This spatially restricted ubiquitination is essential for chromosome biorientation and accurate chromosome alignment, demonstrating that ubiquitination regulates mitotic progression by controlling the spatiotemporal localization of PLK1.

Beyond controlling subcellular localization, ubiquitination is the principal mechanism governing PLK1 protein homeostasis. Multiple E3 ubiquitin ligases, including SKP1–CUL1–F-box (SCF)^β-transducin repeat-containing protein (βTrCP)^ E3 ubiquitin ligase complex, APC/C^Cdh1^, cullin 4 (CUL4)–cereblon (CRBN), and ubiquitin-associated protein 2-like (UBAP2L), promote PLK1 ubiquitination and degradation through distinct regulatory pathways (Giráldez et al., 2014; Giráldez et al., 2017; Wang et al., 2020; Bassermann et al., 2008; Guerber et al., 2023; Li et al., 2020). SCF^βTrCP^ primarily targets PLK1 for proteasomal degradation during the G1/S transition (Giráldez et al., 2014; Giráldez et al., 2017). Whereas APC/C^Cdh1^ mediates PLK1 degradation in response to DNA damage during G2 phase, thereby preventing premature mitotic entry (Wang et al., 2020; Bassermann et al., 2008). PLK1 degradation also contributes to mitotic exit and cytokinesis. Beginning at anaphase onset, PLK1 undergoes progressive, proteasome-dependent degradation requiring a D-box-like motif. Although PLK1 is an APC/C^Cdh1^ substrate, APC/C^Cdh1^ activation alone appears insufficient to initiate its degradation, suggesting additional temporal regulation (Lindon and Pines, 2004). Progressive PLK1 degradation permits centrosomal protein 55 (CEP55) recruitment to the midbody and subsequent abscission (Bastos and Barr, 2010). Integrin–FAK–Src signaling further regulates this process by slowing PLK1 degradation and delaying CEP55 recruitment to the midbody (Kamranvar et al., 2016). UBAP2L facilitates the dynamic removal of PLK1 from multiple mitotic structures by promoting its ubiquitination and degradation, and loss of UBAP2L results in abnormal stabilization of PLK1 (Guerber et al., 2023). Notably, PLK1 has also been identified as a substrate of the CUL4–CRBN E3 ubiquitin ligase complex. In the presence of the AAA ATPase p97, immunomodulatory drugs (IMiDs) induce CUL4–CRBN-dependent degradation of PLK1 (Li et al., 2020).

Ubiquitination also directly regulates cell-cycle checkpoint signaling. The E3 ubiquitin ligase CHFR catalyzes ubiquitination of PLK1 at K209, leading to PLK1 degradation, delayed activation of CDC25C, sustained WEE1 G2 checkpoint kinase (WEE1) activity, inhibition of CDK1 activation, and consequently delayed mitotic entry (Zhu et al., 2021; Kang et al., 2002). Importantly, this pathway is antagonized by VRK2-mediated phosphorylation of T210, providing a representative example of functional crosstalk between phosphorylation and ubiquitination in regulating PLK1 activity (Zhu et al., 2021).

Accumulating evidence indicates that dysregulated PLK1 ubiquitination contributes to tumor development and progression. In contrast to degradative K48-linked ubiquitination, tripartite motif containing 47 (TRIM47)-mediated K63-linked ubiquitination stabilizes PLK1 and enhances its kinase activity, thereby activating the nuclear factor kappa B (NF-κB) and mitogen-activated protein kinase (MAPK) signaling pathways to promote hepatocellular carcinoma cell proliferation (Xie et al., 2025). Likewise, BAG cochaperone 6 (BAG6) maintains PLK1 stability in hepatocellular carcinoma by suppressing its polyubiquitination (Zhang et al., 2026). In prostate cancer cells, C-terminus of HSC70-interacting protein (CHIP)-mediated ubiquitination of PLK1 participates in the DNA damage response under cellular stress. Loss of CHIP permits cells harboring unrepaired DNA damage to continue progressing through the cell cycle, ultimately leading to mitotic catastrophe and cell death (Chen et al., 2016). Together, these findings demonstrate that aberrant ubiquitination not only disrupts PLK1 protein homeostasis but also promotes tumor progression by altering cell proliferation, DNA damage responses, and genome stability.

Collectively, ubiquitination regulates PLK1 through multiple mechanisms, including subcellular localization, protein turnover, cell-cycle checkpoint control, and tumor-associated signaling. Furthermore, its extensive crosstalk with phosphorylation provides an additional regulatory layer that fine-tunes PLK1 activity in a spatiotemporal manner, underscoring the importance of coordinated PTM networks in maintaining mitotic fidelity.

The currently identified PLK1 ubiquitination sites and their regulatory mechanisms are summarized in Table 2.

**TABLE 2:** PLK1 ubiquitination.

E3 ligase/regulator	Ubiquitination site/type	Effect on PLK1	Disease/cell model	Biological outcome
CUL3–KLHL22 (Beck et al., 2013; McGourty and Rape, 2013)	K492 mono-ubiquitination	Promotes PLK1 dissociation from kinetochores without affecting its abundance	Mitotic cells	Ensures chromosome biorientation and accurate chromosome alignment
SCF^βTrCP^ (Giráldez et al., 2014; Giráldez et al., 2017)	Ubiquitination (site ND)	Promotes PLK1 degradation	G1/S-phase cells	Regulates PLK1 turnover during the G1/S transition
APC/C^Cdh1^ (Wang et al., 2020; Bassermann et al., 2008; Lindon and Pines, 2004; Bastos and Barr)	Ubiquitination (site ND)	Promotes PLK1 degradation	G2-phase cells under DNA damage; mitotic cells	Prevents mitotic entry following DNA damage; regulates mitotic exit and cytokinesis by enabling CEP55 recruitment to the midbody
UBAP2L (Guerber et al., 2023)	Ubiquitination (site ND)	Promotes PLK1 degradation	Mitotic cells	Facilitates PLK1 removal from mitotic structures; UBAP2L loss causes aberrant PLK1 stabilization
CUL4–CRBN (Li et al., 2020)	p97-dependent ubiquitination (site ND)	Promotes IMiD-induced degradation of PLK1	IMiD-treated cells	Reduces PLK1 abundance through CUL4–CRBN-dependent degradation
CHFR (Kang et al., 2002)	K209 ubiquitination	Promotes PLK1 degradation	Mitotic cells	Delays CDC25C activation and WEE1 inactivation, thereby suppressing CDK1 activation and delaying mitotic entry
TRIM47 (Xie et al., 2025)	K63-linked ubiquitination (site ND)	Stabilizes PLK1 and enhances its kinase activity	Hepatocellular carcinoma cells	Activates NF-κB and MAPK signaling and promotes tumor cell proliferation
BAG6 (Zhang et al., 2026)	Polyubiquitination (site ND)	Inhibits PLK1 polyubiquitination	Hepatocellular carcinoma cells	Maintains PLK1 stability
CHIP (Chen et al., 2016)	Ubiquitination (site ND)	Promotes PLK1 ubiquitination	Prostate cancer cells	Participates in the DNA damage response; Prevents cell-cycle progression with unrepaired DNA damage

This table summarizes the major regulators, ubiquitination sites/types, effects on PLK1, and biological outcomes of PLK1 ubiquitination. ND, not determined.

### 6.2 PLK1 deubiquitination

Counteracting ubiquitin-mediated degradation, multiple deubiquitinases stabilize PLK1 by removing ubiquitin chains, thereby regulating cell-cycle progression and diverse pathological processes (Squair and Virdee, 2022). To date, numerous deubiquitinating enzymes (DUBs) have been identified as regulators of PLK1, including ubiquitin-specific peptidase 1 (USP1), USP3, USP7, USP10, USP16, USP18, USP24, USP39, and OTU deubiquitinase 3 (OTUD3).

The predominant function of PLK1 deubiquitination is to maintain protein stability by removing K48-linked polyubiquitin chains, thereby preventing proteasomal degradation. Representative examples include USP1, which stabilizes PLK1 to enhance LDHA expression and promote glycolysis and proliferation in T-cell acute lymphoblastic leukemia (T-ALL) (Liu et al., 2023). Similarly, USP3, USP7, USP10, USP18, USP24, and USP39 stabilize PLK1 to promote tumor progression in glioma, prostate cancer, pancreatic ductal adenocarcinoma, esophageal cancer, head and neck squamous cell carcinoma, and gastric cancer by enhancing cell proliferation, activating oncogenic signaling pathways (including mechanistic target of rapamycin complex 1 (mTORC1) and Notch receptor 1 (NOTCH1)), promoting glycolytic metabolism, or conferring therapeutic resistance (Yan et al., 2026; Peng et al., 2019; Du et al., 2025; Ma et al., 2025; Li et al., 2026; Geng et al., 2024; Zhi et al., 2023; Shen et al., 2025). Collectively, maintenance of PLK1 stability through deubiquitination has emerged as a common mechanism driving malignant progression across multiple tumor types.

Beyond cancer, PLK1 deubiquitination also contributes to non-neoplastic diseases. OTUD3 stabilizes PLK1 by removing K48-linked ubiquitin chains and exerts neuroprotective effects during cerebral ischemia–reperfusion injury through regulation of the phosphoinositide 3-kinase (PI3K)/AKT serine/threonine kinase (AKT) signaling pathway, increased glutathione abundance, enhanced GPX4 activity, and suppression of ferroptosis (Cheng et al., 2025).

In addition to regulating protein stability, deubiquitination also controls the spatiotemporal localization of PLK1. USP16 removes ubiquitin from PLK1 and enhances its interaction with kinetochore-associated proteins such as BUBR1, thereby promoting PLK1 retention at kinetochores to ensure accurate chromosome alignment during early mitosis (Zhuo et al., 2015). Together, USP16-mediated deubiquitination and CUL3–KLHL22-mediated mono-ubiquitination establish a reversible ubiquitination cycle that precisely controls PLK1 kinetochore dynamics during the prometaphase-to-metaphase transition (Zhuo et al., 2015).

Interestingly, deubiquitination itself is subject to regulation by other PTMs. O-GlcNAcylation of USP16 enhances its deubiquitinating activity toward PLK1, thereby promoting faithful chromosome segregation during mitosis (Zhao et al., 2024). This finding provides another representative example of functional crosstalk between O-GlcNAcylation and ubiquitination in the regulation of PLK1.

Collectively, deubiquitination regulates PLK1 through multiple mechanisms, including maintenance of protein stability, control of subcellular localization, and coordination with other post-translational modifications. These regulatory processes collectively contribute to cell-cycle progression, tumor development, and diverse physiological and pathological functions.

The currently identified deubiquitinases that regulate PLK1 and their biological functions are summarized in Table 3.

**TABLE 3:** PLK1 deubiquitinase.

Deubiquitinase (DUB)	Target ubiquitination event	Effect on PLK1	Disease/cell model	Biological outcome
USP1 (Liu et al., 2023)	Deubiquitination of PLK1	Stabilizes PLK1	T-cell acute lymphoblastic leukemia (T-ALL)	PLK1–LDHA signaling; glycolysis and proliferation
USP3 (Yan et al., 2026)	Removes K48-linked polyubiquitin chains	Stabilizes PLK1	Glioma	Cell proliferation and invasion
USP7 (Peng et al., 2019)	Deubiquitinates PLK1 (USP7 catalytic residue C223 is required)	Stabilizes PLK1	Prostate cancer (DU145, VCaP)	Paclitaxel resistance; mitotic progression
USP10 (Du et al., 2025)	Deubiquitinates PLK1	Stabilizes PLK1	Pancreatic ductal adenocarcinoma (PDAC)	Autophagy; gemcitabine resistance
USP10 (Ma et al., 2025)	Deubiquitinates PLK1	Stabilizes PLK1	Esophageal cancer	YTHDC1–USP10–PLK1 axis; tumor progression and radioresistance
USP10 (Li et al., 2026)	Removes K48-linked polyubiquitin chains	Stabilizes PLK1	Glioma	Proliferation, migration, and apoptosis inhibition
USP16 (Zhuo et al., 2015)	Reverses CUL3-mediated ubiquitination at PLK1 K492	Promotes PLK1 kinetochore localization	HeLa cells	Chromosome alignment and segregation
USP18 (Geng et al., 2024)	Removes K48-linked polyubiquitin chains	Stabilizes PLK1	Head and neck squamous cell carcinoma (HNSCC)	mTORC1 activation; proliferation, migration and invasion
USP24 (Zhi et al., 2023)	Deubiquitinates PLK1	Stabilizes PLK1	Gastric cancer	NOTCH1 activation; glycolysis
USP39 (Shen et al., 2025)	Deubiquitinates PLK1	Stabilizes PLK1	Prostate cancer	Tumor progression; apoptosis and ferroptosis suppression
OTUD3 (Cheng et al., 2025)	Removes K48-linked polyubiquitin chains	Stabilizes PLK1	Cerebral ischemia/reperfusion injury	PI3K-AKT activation; ferroptosis inhibition

This table summarizes the currently identified deubiquitinases (DUBs) involved in PLK1 regulation, highlighting their target ubiquitination sites or events, effects on PLK1 protein stability and activity, experimental models, and associated biological consequences.

## 7 PLK1 SUMOylation

SUMOylation is a reversible post-translational modification mediated by small ubiquitin-like modifier (SUMO) proteins that regulates protein localization, stability, and protein–protein interactions (Flotho and Melchior, 2013; Chang and Yeh, 2020; Yeh, 2009). Although studies on PLK1 SUMOylation remain limited, accumulating evidence indicates that SUMOylation plays important roles in controlling PLK1 subcellular localization, protein stability, and cell division.

One of the major functions of SUMOylation is to regulate the spatiotemporal localization of PLK1. During mouse oocyte meiosis, PLK1 is modified by both SUMO-1 and SUMO-2/3, which exhibit distinct localization patterns. SUMO-1-modified PLK1 is predominantly localized to the spindle and spindle poles in a spindle-dependent manner, whereas SUMO-2/3-modified PLK1 is enriched at kinetochores independently of spindle integrity (Feitosa et al., 2018). This regulatory mechanism appears to be evolutionarily conserved. In the silkworm, the suppressor of Mif Two 3 (Smt3)/ubiquitin-conjugating enzyme 9 (Ubc9) complex mediates SUMOylation of PLK1 at K466, corresponding to conserved lysine residues in *Drosophila* DmPolo (K473) and human PLK1 (K492) (Li et al., 2017). Disruption of SUMOylation results in aberrant localization of PLK1 at centrosomes, kinetochores, and chromosome-associated regions, ultimately impairing chromosome alignment and segregation (Li et al., 2017). These findings demonstrate that SUMOylation is essential for the precise spatiotemporal distribution of PLK1 during both mitosis and meiosis.

In addition to regulating subcellular localization, SUMOylation also contributes to PLK1 protein stability. Ubc9-mediated SUMOylation at K492 promotes nuclear import of PLK1 and prevents APC/C^Cdh1^-mediated ubiquitin-dependent degradation during mitosis by weakening the interaction between PLK1 and APC/C^Cdh1^, thereby maintaining PLK1 protein stability (Wen et al., 2017). These findings further suggest a functional interplay between SUMOylation and ubiquitination, in which SUMOylation antagonizes ubiquitin-mediated degradation to fine-tune PLK1 turnover.

Beyond regulating PLK1 localization and stability, SUMOylation is also essential for PLK1-mediated meiotic progression. In mouse oocytes, K178 and K191 have been identified as major SUMOylation sites. Aberrant hyper-SUMOylation of PLK1 disrupts spindle organization, alters PLK1 localization, and reduces first polar body extrusion, indicating that precise regulation of SUMOylation is required for normal meiotic progression (Mo et al., 2022). Notably, during metaphase II (MII), phosphorylated PLK1 at T210 and S137 colocalizes with SUMO-1 and SUMO-2/3, respectively, suggesting functional cooperation between SUMOylation and phosphorylation in regulating chromosome attachment and segregation (Feitosa et al., 2018).

Evidence also indicates that dysregulated SUMOylation may contribute to reproductive aging. In aged mouse oocytes, PLK1 expression is increased, whereas SUMO-1 and SUMO-2/3 levels are markedly reduced, implying that impaired PLK1 SUMOylation may be associated with age-related meiotic defects (Mo et al., 2022). More recent studies further demonstrated that PLK1 SUMOylation is required not only during oocyte meiosis but also during spermatocyte meiosis, where disruption of SUMOylation impairs meiotic progression and alters PLK1 kinase activity (Xiao et al., 2017; Applebaum et al., 2023).

Collectively, these findings demonstrate that SUMOylation regulates PLK1 through multiple mechanisms, including subcellular localization, protein stability, and meiotic progression. Moreover, its functional interplay with phosphorylation and ubiquitination further highlights the complexity of PLK1 post-translational regulation and supports the concept that coordinated PTM crosstalk is essential for the precise spatiotemporal control of PLK1 activity.

## 8 PTM crosstalk orchestrates PLK1 function

Increasing evidence indicates that PTMs do not regulate PLK1 independently but instead form an integrated regulatory network through cooperative or antagonistic interactions. These PTM crosstalk mechanisms collectively determine PLK1 activity, protein stability, subcellular localization, and mitotic function, thereby enabling precise spatiotemporal control of PLK1 during cell-cycle progression.

The best-characterized crosstalk occurs between phosphorylation and ubiquitination. CHFR mediates ubiquitination of PLK1 at K209, leading to PLK1 degradation and delayed mitotic entry through inhibition of CDK1 activation (Zhu et al., 2021; Kang et al., 2002). Conversely, T210 phosphorylation, mediated by VRK2, disrupts the interaction between PLK1 and CHFR, thereby antagonizing K209 ubiquitination and stabilizing PLK1 (Zhu et al., 2021).

Similarly, c-Abl-mediated phosphorylation at Y425 promotes Aurora A-dependent phosphorylation of T210 while suppressing ubiquitin-dependent degradation, further enhancing PLK1 activation and ensuring timely mitotic entry (Yang et al., 2017). In contrast, PKMYT1-mediated phosphorylation of Y217 inhibits T210 phosphorylation, thereby restraining PLK1 kinase activity (Caron et al., 2016). Collectively, these findings suggest that phosphorylation regulates not only PLK1 activity but also its protein turnover through coordinated modulation of ubiquitination.

Notably, methylation and phosphorylation of PLK1 form a reciprocal regulatory circuit. G9a-mediated methylation of K209 antagonizes Aurora A-dependent phosphorylation of T210, whereas phosphorylation of T210 reciprocally prevents K209 methylation, together establishing a dynamic molecular switch that fine-tunes PLK1 activation throughout the cell cycle (Li et al., 2019). This reciprocal regulatory switch provides an additional mechanism for dynamically balancing PLK1 activation during cell-cycle progression.

Distinct from these antagonistic interactions, O-GlcNAcylation and SUMOylation cooperate with other PTMs to regulate PLK1 stability and localization. Unlike the canonical role of O-GlcNAcylation in protecting proteins from ubiquitin-mediated degradation, O-GlcNAcylation of PLK1 promotes ubiquitination, thereby facilitating protein turnover and proper mitotic progression (Yan et al., 2023; Wang et al., 2010; Qin et al., 2017). By contrast, SUMOylation at K492 antagonizes APC/C^Cdh1^-mediated ubiquitination, stabilizing PLK1 and promoting its nuclear localization (Wen et al., 2017). Moreover, phosphorylated PLK1 at T210 and S137 colocalizes with SUMO-1 and SUMO-2/3, respectively, during meiosis II, suggesting functional cooperation between SUMOylation and phosphorylation in chromosome attachment and segregation (Feitosa et al., 2018). Consistent with this notion, dysregulated SUMOylation has been associated with altered PLK1 kinase activity and impaired meiotic progression, although the molecular basis underlying this interaction remains to be elucidated (Xiao et al., 2017; Applebaum et al., 2023).

Collectively, current evidence supports a model in which phosphorylation serves as the central regulatory axis of PLK1, while ubiquitination, methylation, SUMOylation, and O-GlcNAcylation function cooperatively or antagonistically to fine-tune PLK1 activation, stability, localization, and downstream signaling. Although only a limited number of PTM interactions have been experimentally characterized, these findings highlight PTM crosstalk as a fundamental mechanism underlying the precise spatiotemporal regulation of PLK1. Further elucidation of this integrated regulatory network may provide new opportunities for targeting PLK1 in cancer therapy.

The major crosstalk mechanisms among the five principal PTMs of PLK1 are summarized in Figure 2.

**FIGURE 2:**
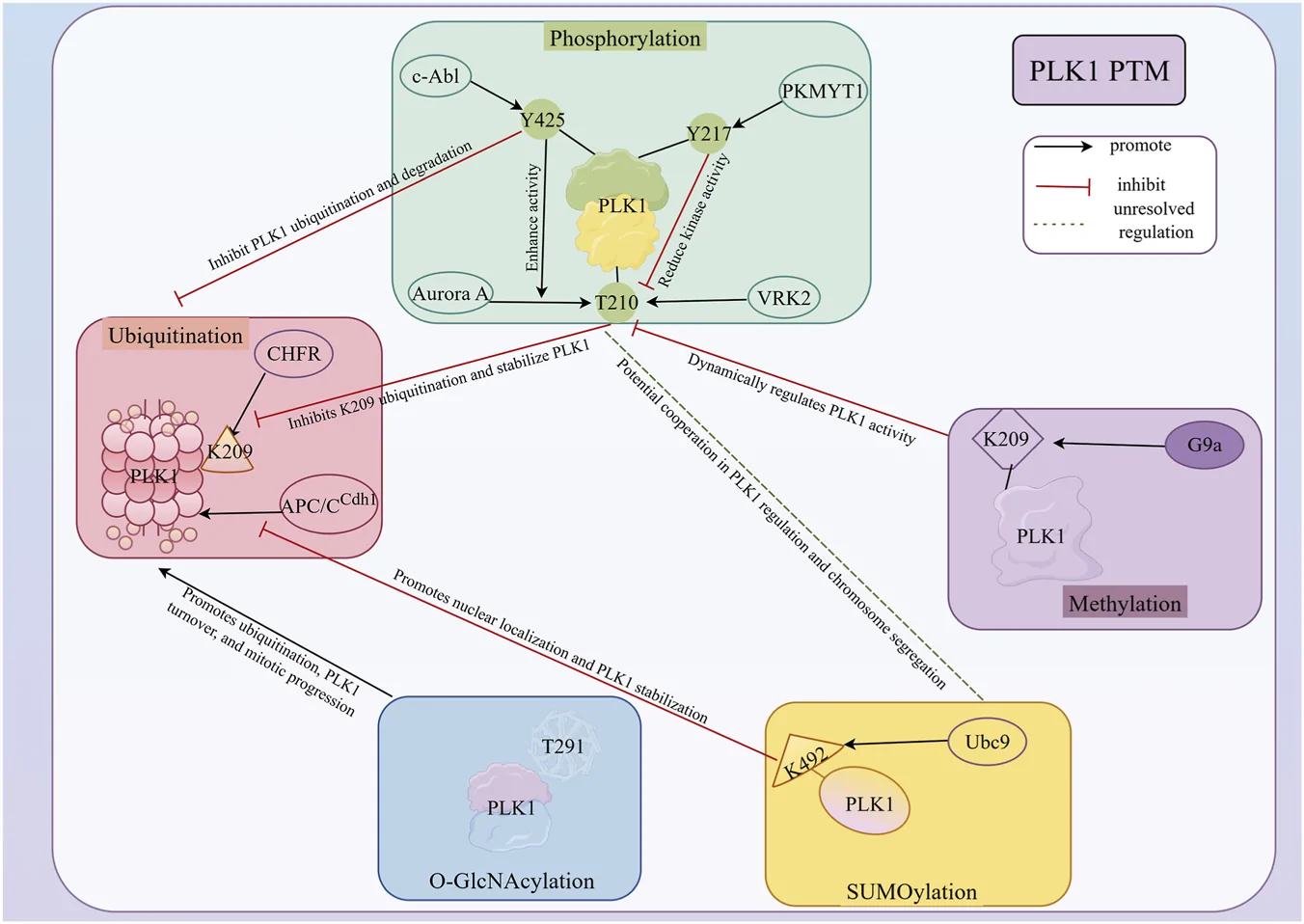
Crosstalk among the five major post-translational modifications of PLK1. Site-specific phosphorylation constitutes the core regulatory network of PLK1, in which distinct phosphorylation events reciprocally influence one another to either promote or restrain PLK1 activation. Phosphorylation also stabilizes PLK1 by antagonizing ubiquitin-mediated degradation. Likewise, SUMOylation and O-GlcNAcylation enhance PLK1 stability by suppressing ubiquitination. Methylation negatively regulates PLK1 phosphorylation and consequently inhibits PLK1 activation. Although functional crosstalk between SUMOylation and phosphorylation has been proposed, the underlying mechanisms remain unclear. Together, these PTMs form an integrated regulatory network that fine-tunes PLK1 activation, stability, localization, and biological functions (By Figdraw).

## 9 Conclusion and perspectives

In this review, we systematically summarized the current understanding of the post-translational modifications (PTMs) of Polo-like kinase 1 (PLK1) and their roles in regulating cell-cycle progression and tumor development. Phosphorylation, ubiquitination, SUMOylation, methylation, and O-GlcNAcylation collectively regulate PLK1 kinase activity, protein stability, subcellular localization, and protein interactions, thereby ensuring the precise spatiotemporal control of PLK1 throughout the cell cycle.

An emerging concept from recent studies is that PTMs function as an integrated regulatory network rather than as independent modifications. Crosstalk among phosphorylation, ubiquitination, SUMOylation, methylation, and O-GlcNAcylation dynamically coordinates PLK1 activation, protein turnover, and mitotic progression, providing an additional layer of regulatory complexity. Although only a limited number of PTM crosstalk mechanisms have been experimentally characterized, current evidence highlights their central role in fine-tuning PLK1 function under both physiological and pathological conditions.

Despite substantial progress, important questions remain regarding the upstream regulatory enzymes, the temporal and spatial coordination of PTMs, and how dysregulated PTM networks contribute to tumor progression and therapeutic resistance. Future advances in quantitative proteomics, structural biology, single-cell and spatial multi-omics, and artificial intelligence-assisted protein modeling are expected to accelerate the systematic characterization of PLK1 PTM networks. A more comprehensive understanding of PTM crosstalk will not only advance our knowledge of PLK1 biology but also facilitate the identification of novel biomarkers and therapeutic strategies for human cancers.

## Glossary

c-Abl: ABL proto-oncogene 1, non-receptor tyrosine kinase
APC/C: anaphase-promoting complex/cyclosome
ATR: ATM and Rad3-related
ATM: ataxia telangiectasia mutated
BAG6: BAG cochaperone 6
βTrCP: β-transducin repeat-containing protein
BUBR1: budding uninhibited by benzimidazole-related 1
Cdh1: Cdc20 homolog 1
CDC25C: cell division cycle 25C
CHIP: C-terminus of HSC70-interacting protein
CHK2: checkpoint kinase 2
CHFR: checkpoint with forkhead and ring finger domains
CIN: chromosomal instability
CUL3: cullin 3
CUL4: cullin 4
CDK1: cyclin-dependent kinase 1
DSBs: DNA double-strand breaks
DAB2IP: Disabled homolog 2-interacting protein
EMT: epithelial–mesenchymal transition
ERK: extracellular signal-regulated kinase
EYA: EYA transcriptional coactivator and phosphatase
EHMT2: euchromatic histone lysine methyltransferase 2, also known as G9a
IMiDs: immunomodulatory drugs
KD: kinase domain
KLHL22: kelch-like family member 22
KAT7: lysine acetyltransferase 7
HBO1: histone acetyltransferase binding to ORC1
mTORC1: mechanistic target of rapamycin complex 1
MII: metaphase II
MTOCs: microtubule-organizing centers
MAPK: mitogen-activated protein kinase
MEK1/2: mitogen-activated protein kinase 1/2
MPS1: monopolar spindle 1
MYPT1: myosin phosphatase target subunit 1
NF-κB: nuclear factor kappa B
NLP: ninein-like protein
NOTCH1: Notch receptor 1
OGT: O-linked N-acetylglucosamine transferase
Op18: oncoprotein 18
OTUD3: OTU deubiquitinase 3
PI3K-AKT: phosphoinositide 3-kinase (PI3K)/AKT serine/threonine kinase (AKT) signaling pathway
PIM2: Pim-2 proto-oncogene, serine/threonine kinase
PLK1: Polo-like kinase 1
PBD: polo-box domain
PBIP1: polo-box-interacting protein 1
PICH: polo-like kinase 1-interacting checkpoint helicase
PAR: poly (ADP-ribose)
PTMs: post-translational modifications
PKA: protein kinase A
PKMYT1: protein kinase, membrane-associated tyrosine/threonine 1
PP1cβ: protein phosphatase 1 catalytic subunit beta
PP2A: protein phosphatase 2A
PPP4C: protein phosphatase 4 catalytic subunit
RAD51: RAD51 recombinase
MK2: MAP kinase-activated protein kinase 2
CEP55: centrosomal protein 55
